# Supplementary material for: From sequence to enzyme mechanism using multi-label machine learning
Source: BMC Bioinformatics. 2014 May 19;15:150. doi: 10.1186/1471-2105-15-150 (PMC4229970; doi:10.1186/1471-2105-15-150)
Supplement: Additional file 2 — Java code of ml2db. Additional file ml2db_code.tar.gz contains the Java source code to run the multi-label machine learning experiments and save the results to database. The code’s Javadoc is included. [file 1471-2105-15-150-S2.zip › additional file 2/ml2db/ml2db/doc/mulan/classifier/transformation/TransformationBasedMultiLabelLearner.html]

TransformationBasedMultiLabelLearner


JavaScript is disabled on your browser.


- Overview
- Package
- Class
- Use
- Tree
- Deprecated
- Index
- Help

- Prev Class
- Next Class

- Frames
- No Frames

- All Classes

- Summary:
- Nested |
- Field |
- Constr |
- Method

- Detail:
- Field |
- Constr |
- Method


mulan.classifier.transformation

## Class TransformationBasedMultiLabelLearner

- java.lang.Object
- - mulan.classifier.MultiLabelLearnerBase
  - - mulan.classifier.transformation.TransformationBasedMultiLabelLearner

- All Implemented Interfaces:
  :   java.io.Serializable, MultiLabelLearner, weka.core.TechnicalInformationHandler

  Direct Known Subclasses:
  :   BinaryRelevance, CalibratedLabelRanking, ClassifierChain, EnsembleOfClassifierChains, EnsembleOfPrunedSets, IncludeLabelsClassifier, LabelPowerset, MultiClassLearner, MultiLabelStacking

  ---

    

  ```
  public abstract class TransformationBasedMultiLabelLearner
  extends MultiLabelLearnerBase
  ```

  Version:
  :   2010.12.25

  Author:
  :   Robert Friberg, Jozef Vilcek, Grigorios Tsoumakas

  See Also:
  :   Serialized Form

- - ### Constructor Summary

    Constructors

    Constructor and Description

    `TransformationBasedMultiLabelLearner()`

    Creates a new instance of `TransformationBasedMultiLabelLearner`
    with default
    `J48` base classifier.

    `TransformationBasedMultiLabelLearner(weka.classifiers.Classifier baseClassifier)`

    Creates a new instance.
  - ### Method Summary

    Methods

    | Modifier and Type | Method and Description |
    | --- | --- |
    | `weka.classifiers.Classifier` | `getBaseClassifier()` Returns the `Classifier` which is used internally by the learner. |
    | `weka.core.TechnicalInformation` | `getTechnicalInformation()` Returns an instance of a TechnicalInformation object, containing detailed information about the technical background of this class, e.g., paper reference or book this class is based on. |
    | `java.lang.String` | `globalInfo()` Returns a string describing the classifier. |

    - ### Methods inherited from class mulan.classifier.MultiLabelLearnerBase

      `build, getDebug, isUpdatable, makeCopy, makePrediction, setDebug`
    - ### Methods inherited from class java.lang.Object

      `equals, getClass, hashCode, notify, notifyAll, toString, wait, wait, wait`

- - ### Constructor Detail


    - #### TransformationBasedMultiLabelLearner

      ```
      public TransformationBasedMultiLabelLearner()
      ```

      Creates a new instance of `TransformationBasedMultiLabelLearner`
      with default
      `J48` base classifier.


    - #### TransformationBasedMultiLabelLearner

      ```
      public TransformationBasedMultiLabelLearner(weka.classifiers.Classifier baseClassifier)
      ```

      Creates a new instance.

      Parameters:
      :   `baseClassifier` - the base classifier which will be used internally
          to handle the data.

      See Also:
      :   `Classifier`
  - ### Method Detail


    - #### getBaseClassifier

      ```
      public weka.classifiers.Classifier getBaseClassifier()
      ```

      Returns the `Classifier` which is used internally by the learner.

      Returns:
      :   the internally used classifier


    - #### getTechnicalInformation

      ```
      public weka.core.TechnicalInformation getTechnicalInformation()
      ```

      Returns an instance of a TechnicalInformation object, containing detailed
      information about the technical background of this class, e.g., paper
      reference or book this class is based on.

      **Specified by:**
      :   `getTechnicalInformation` in interface `weka.core.TechnicalInformationHandler`

      **Specified by:**
      :   `getTechnicalInformation` in class `MultiLabelLearnerBase`

      Returns:
      :   the technical information about this class


    - #### globalInfo

      ```
      public java.lang.String globalInfo()
      ```

      Returns a string describing the classifier.

      **Specified by:**
      :   `globalInfo` in class `MultiLabelLearnerBase`

      Returns:
      :   a string description of the classifier


- Overview
- Package
- Class
- Use
- Tree
- Deprecated
- Index
- Help

- Prev Class
- Next Class

- Frames
- No Frames

- All Classes

- Summary:
- Nested |
- Field |
- Constr |
- Method

- Detail:
- Field |
- Constr |
- Method
